# Targeted approaches to improve outcomes for highest-cost patients

**DOI:** 10.1186/s13584-017-0151-6

**Published:** 2017-06-07

**Authors:** Adam J. Rose

**Affiliations:** Department of Medicine, Section of General Internal Medicine, Boston University School of Medicine, 801 Massachusetts Avenue, 2nd Floor, Boston, MA 02118 USA

## Abstract

Bash and colleagues, using data from Maccabi Healthcare Services, have documented increased cost and utilization attributable to patients with congestive heart failure (CHF). The CHF patients were older than the general population and had high rates of important comorbid conditions. While it is somewhat predictable that such a population would have higher healthcare utilization and costs, the extent of the difference was still surprising. Most CHF patients (78%) were hospitalized at least once, compared to only 21% of patients without CHF. CHF patients used dramatically more of every kind of health care, including physician visits, emergency department visits, and specialty care visits. In this paper, Bash and colleagues have provided essential information about the “cost epidemiology” of CHF patients in the Israeli context.

This commentary places these results in a broader context of how “cost epidemiology” information can be translated into targeted programs to improve outcomes and costs for vulnerable populations. The commentary makes three key points. First, beyond showing the increased utilization and cost attributable to CHF, there is also a need to examine which patients within this broad category contribute most to these increased costs, and might therefore be targeted for enhanced services. Second, it is helpful to make a business case for intervening to improve outcomes with a subpopulation, focusing in particular on the return on investment from the standpoint of the payer. Finally, while Israeli health collectives have already deployed programs to improve outcomes in older and sicker patients, there may be a need to more precisely define important subpopulations based on social risk factors or particularly severe disease manifestations, and then target those subpopulations with tailored programs focused on their particular needs.

## “Cost epidemiology” of congestive heart failure in an Israeli context

Bash and colleagues have examined an important subpopulation of Israeli patients – those with newly diagnosed congestive heart failure (CHF) [1]. Using the powerful database of Maccabi Healthcare Services (MHS), the authors show that CHF patients were older than the general population and had high rates of important comorbid conditions. While it is somewhat predictable that such a population would have higher healthcare utilization and costs, the extent of the difference was still surprising. Most CHF patients (78%) were hospitalized at least once, compared to only 21% of patients without CHF. CHF patients used dramatically more of every kind of health care, including physician visits, emergency department visits, and specialty care visits. Their overall cost of care was many-fold higher.

## Using local “cost epidemiology” as a basis for planning interventions

The finding that CHF patients are a high-cost group, due in large part to the costs of hospital care, is not a new finding [2]. What is new here is that there is great policy value for Israeli health care managers in knowing the up-to-date details about which populations are contributing to healthcare costs the most – in the specific context of a large Israeli health plan. A further investigation would be to examine predictors of cost within this population of Israeli CHF patients, to see how well we can predict which patients have the greatest need, and therefore would benefit most from targeted services. These drivers of cost and outcomes may not be merely biological, but may also relate to mental health conditions, social situation, poverty, and differential access to care. The present study by Bash et al, then, can properly be seen as “cost epidemiology”, which needs to be followed by further health services research, and finally by an intervention built upon those results.

The idea of establishing programs to improve care for vulnerable subpopulations is not new, but it is a rapidly evolving field. Many of the prominent studies of case management or other related approaches have taken place in the United States [3], a nation whose healthcare challenges are very different from those of Israel. Israel does have underserved populations, including rural dwellers, poorer residents, and certain ethnic minorities such as Israeli Arabs or Russian speakers. However, the social challenges of providing healthcare in the United States are unequalled, and it has been posited that it is the unique degree of concentrated and persistent poverty in the United States that drives the exceptionalism of higher costs and poorer outcomes [4]. Given these differences, there may be limited value in Israeli health collectives patterning programs after successful efforts to address the highest cost patients in the United States [5]. However, what can be gleaned from prior successful efforts is that they have all begun with a thorough understanding of the problem to be addressed – including not only the patients and their biopsychosocial realities, but also the capabilities of the local system to help them under present conditions. Thus, the present study by Bash and colleagues provides a valuable starting point for understanding one highly vulnerable group of Israeli patients.

## Making a “business case” for an intervention

In addition to understanding the scope of the problem, and designing an intervention that is likely to help, there is a third important step – namely, securing the necessary political consensus to act. One way that health policy researchers can help build a case for action is through the judicious use of a business case analysis. A business case analysis uses the familiar tools of cost-benefit and simulation analyses, but generally disregards improvement in health-related quality of life and focuses exclusively on the amount that might be saved through various levels of improvement. We have completed several business case analyses of this sort, which have indeed helped build a case for concrete changes within an integrated health system not unlike an Israeli health collective. In one example, we examined the amount of money that the Veterans Health Administration (VA) could save by improving the population level of control with warfarin, a commonly used anticoagulant [6]. We demonstrated the potential cost savings from averted adverse events (bleeding, strokes, etc.) with varying levels of potential improvement. We left unexamined the question of how much improvement could be achieved or how much it would cost to do so; quality-adjusted life year gains were calculated but not considered for the purpose of the business case analysis. On the basis of these findings, VA invested in a pilot program to improve warfarin management [7], and the success of this pilot led to the spread of this approach across the entire VA system. One can imagine the results of Bash and colleagues forming the basis for a similar business case analysis of how much MHS could save through decreasing hospital admissions among CHF patients to varying degrees. The business case analysis could be even stronger if a subgroup of CHF patients could first be identified who have even higher risk and even higher costs.

## Existing programs to improve outcomes for vulnerable subpopulations: what has been accomplished, and what might be improved?

The leadership at MHS, of course, are well aware of the potential savings from improving care for vulnerable subgroups. They have recently introduced three relevant programs to improve outcomes for vulnerable subgroups, including CHF patients:

1. Universal outreach following hospital discharge: In 2015, MHS introduced the Maccabi Transitional Care Program (MTCP), which involves an attempt to contact every patient by telephone following hospital discharge and facilitate needed care. These patients vary greatly in illness burden, discharge diagnosis, and level of need, but it is unquestionably true that the period following hospital discharge is a high-risk period even for patients who are neither sick nor needy.
2. High-Intensity Case Management for Complex Community-Dwelling Elders: Also in 2015, MHS introduced the Community Program, which focused on intensive case management for community-dwelling elders with multimorbidity. This represents a higher level of intensity in case management, and is targeted at a smaller population in an ongoing way.
3. Telemedicine: This program, begun in 2013, combines telemedicine monitoring with protocol-based disease management, for chronically ill patients, including those with specific conditions such as severe CHF or chronic lung disease.

These programs are predicated upon other streams of epidemiology research, such as a study that showed that fully two thirds of MHS patients have multimorbidity, defined as two or more chronic conditions [8]. Also, MHS is not the only Israeli health collective to have deployed programs to manage the most vulnerable patients. For example, Clalit, the largest health collective, has a similar initiative called the Comprehensive Care for Multimorbid Adults Program (CC-MAP).

Evaluation will be important to understand the impact these programs are having on outcomes and costs. It may be that these programs can still be further differentiated to focus on more precisely targeted populations, and have an even bigger impact. There are multiple levels on which disease management programs should be differentiated for maximal impact. A specific diagnosis, such as CHF as examined by Bash et al, is only one dimension. Highly successful programs to improve outcomes and decrease costs in highly vulnerable patients have also incorporated a nuanced understanding of the social situation of the target population [9]. Even in a smaller country like Israel, there are many distinct subpopulations that may require particular approaches. There are likely to be particular groups whose risk of poor outcomes or high costs is driven at least as much by their social situation (ethnicity, living situation, etc.) as by any one severe diagnosis (e.g. CHF), a confluence of multiple diagnoses, or even a one-time event such as a recent hospital discharge.

A broad program such as MTCP is a valuable baseline level of service, and evaluation may show that it is worthwhile to provide at least this level of service to everyone discharged from the hospital. However, there may be room for other, more targeted programs. It is possible that the program for community-dwelling elders with multimorbidity may be enhanced by tailoring it to the needs of important sub-populations, such as Arab Israelis or Russian speakers. It is possible that while the general telemedicine program does indeed help improve outcomes for CHF patients, it may be worthwhile to establish a separate program just for them, because they may have very particular needs that are not completely addressed by the general program.

## Conclusions

MHS has recently created several programs to improve outcomes and reduce costs among its most vulnerable patients. Evaluation of these programs will be important to show that they have done some good. However, the possibility remains that we could do even better with a more precisely targeted approach. The “cost epidemiology” information provided by Bash and colleagues about patients with CHF may help managers to more precisely target such programs.

## Abbreviations

CC-MAP: Clalit comprehensive care for multimorbid adults program
CHF: Congestive heart failure
MHS: Maccabi healthcare services
MTCP: Maccabi transitional care program
VA: Veterans health administration of the United States
